# Effects of Drying Methods on Flavor Substances and Identification of Characteristic Flavor Compounds of Dried Tangerine Peel

**DOI:** 10.3390/foods15152669

**Published:** 2026-07-29

**Authors:** Mingxuan Zhang, Minghui Xu, Renpu Wei, Yujie Lu, Huimin Chen, Juan Kan, Man Zhang, Lixia Xiao, Chunlu Qian

**Affiliations:** 1 Department of Food Science and Engineering, School of Food Science and Engineering, Yangzhou University, Yangzhou 225127, China; kenkirk050301@gmail.com (M.Z.); mhui0427@163.com (M.X.); 15895162850@163.com (R.W.); m15052520793_1@163.com (Y.L.); 18068876582@163.com (H.C.); 2 Department of Horticulture, College of Horticulture and Landscape Architecture, Yangzhou University, Yangzhou 225009, China; kanjuan@yzu.edu.cn (J.K.); mzhang100@yzu.edu.cn (M.Z.); lxxiao@yzul.edu.cn (L.X.)

**Keywords:** citrus peel, drying methods, free amino acids, volatile flavor compounds

## Abstract

Drying method is a key factor influencing the nutritional quality and flavor characteristics of Chenpi. In this study, peels of *Citrus reticulata* ‘Chachi’ (Xiaoqinggan) were subjected to natural air drying (NAD), hot-air drying (HAD), and vacuum drying (VD) to evaluate changes in physicochemical properties, free amino acids, and volatile compounds. VD exhibited superior retention of total flavonoids and soluble sugars, resulting in the best overall physicochemical quality. NAD preserved the highest total free amino acid content and amino acid diversity, whereas HAD was characterized by a predominance of bitter amino acids, contributing to a pronounced bitter taste. In contrast, VD maintained a more balanced amino acid composition with relatively high proportions of sweet amino acids and effective retention of aroma precursors. A total of 75 volatile compounds were identified by GC–MS, with olefins representing the dominant class. NAD exhibited the highest total volatile content, while VD retained the greatest diversity of volatile compounds and a more balanced aroma composition. Odor activity value (OAV) analysis revealed that key aroma-active compounds were effectively preserved in VD-treated samples, with linalool identified as the major contributor to citrus peel aroma. Overall, VD achieved the most favorable balance between nutritional quality, taste characteristics, and aroma preservation. These findings provide scientific guidance for optimizing drying technologies and improving the quality of Chenpi products.

## 1. Introduction

Citrus (*Citrus* spp.) is an evergreen small tree of the *Rutaceae* family, Citrus genus. It is widely favored worldwide for its rich flavor and abundant nutrition, and is also one of the economic fruit trees with the largest cultivation area and highest yield in China. Its annual output has ranked first in the world for many consecutive years, with outstanding industrial scale and economic value. In addition to the edible pulp, which is rich in vitamins, minerals, and dietary fiber, citrus peel contains abundant bioactive compounds, including flavonoids, polyphenols, volatile oils, and polysaccharides. These constituents make citrus peel the primary raw material for dried tangerine peel (Chenpi), a traditional medicinal and edible product widely used in China. Owing to its long history of application in tea preparation, culinary seasoning, and healthcare products, Chenpi represents an important avenue for the high-value utilization of citrus by-products [1].

Citrus is a typical seasonal fruit, with a concentrated harvest period from October to December every year. Fresh fruits have high water content and short shelf life, and are extremely perishable. Therefore, drying has become a key link in the processing, storage and quality formation of Chenpi [2]. Traditional Chenpi processing is mainly based on natural air drying. Although it is simple to operate and low in cost, it has a long drying cycle, is greatly affected by environmental weather, and the product quality stability is difficult to guarantee [3].

With the development of modern food processing technology, technologies such as hot air drying, vacuum drying, freeze drying and short-wave infrared drying have been gradually applied in the field of fruit and vegetable drying [4]. Existing studies have shown that freeze drying and short-wave infrared drying have obvious advantages in maintaining the raw material tissue structure and retaining active substances, but they are limited by high equipment investment, large energy consumption and limited continuous production capacity, making it difficult to be widely used in the large-scale production of Chenpi [5].

In this context, three widely applicable drying methods—natural air drying (NAD), hot-air drying (HAD), and vacuum drying (VD)—were selected for comparative investigation. NAD removes moisture under ambient temperature and natural airflow conditions and is suitable for large-scale processing. HAD achieves rapid dehydration through heated airflow, providing high drying efficiency but potentially causing degradation of heat-sensitive compounds. In contrast, VD operates under reduced pressure and relatively low temperatures, which can suppress oxidative reactions and facilitate the retention of nutritional and flavor-related constituents.

At present, there has been a certain accumulation of research on the effects of drying conditions on citrus peel quality at home and abroad, but the existing results still have obvious limitations: most studies focus on the effect of a single drying temperature on the overall flavor or active components of citrus peel, lacking a systematic analysis of the quality change law under different drying mechanisms [6]. Furthermore, existing studies have mainly emphasized the extraction and application of citrus peel bioactive compounds, whereas the coordinated changes in physicochemical properties, taste-related substances, and aroma-active compounds during drying have received comparatively less attention [7,8]. Studies integrating physicochemical characterization, free amino acid profiling, GC–MS analysis, odor activity value (OAV) assessment, and multivariate statistical analysis to comprehensively elucidate the effects of drying methods on Chenpi flavor quality remain scarce [9,10,11].

Therefore, this study systematically investigated the effects of NAD, HAD, and VD on the physicochemical properties, free amino acid composition, and volatile flavor compounds of *Citrus reticulata* ‘Chachi’ peel. High-performance liquid chromatography (HPLC) was employed to characterize non-volatile flavor substances, while GC–MS combined with multivariate statistical analysis was used to evaluate volatile compounds and their variation patterns. Key aroma-active compounds were further identified using the combined criteria of odor activity value (OAV > 1) and variable importance in projection (VIP > 1) [12,13,14]. The objective of this work was to clarify the mechanisms through which different drying methods influence the nutritional and flavor quality of citrus peel, identify the quality characteristics associated with each drying process, and provide scientific guidance for Chenpi processing optimization, flavor regulation, and industrial standardization [15,16].

## 2. Materials and Methods

### 2.1. Experimental Materials and Reagents

The fresh fruits of *Citrus reticulata* ‘Chachi’ used in the experiment were collected from Xinhui District, Jiangmen City, Guangdong Province. After harvest, the pulp was removed immediately, and the fresh citrus peel was taken as the experimental raw material and stored at 4 °C for later use.

Chemical reagents such as rutin, gallic acid, glucose, citric acid, 17 amino acid mixed standards, o-phthalaldehyde (OPA), 2-octanol internal standard, as well as chromatographic pure reagents such as methanol, acetonitrile and n-hexane, and other analytical pure reagents were purchased from Sinopharm Chemical Reagent Co., Ltd. (Shanghai, China); the experimental water was ultrapure water.

### 2.2. Experimental Methods

#### 2.2.1. Sample Drying Treatment

Fresh citrus peels from the same harvest batch were thoroughly mixed and randomly divided into three drying treatments. For each drying treatment, three independent processing replicates were prepared and dried separately. Each replicate was analyzed independently for physicochemical properties, free amino acids, and volatile compounds. During drying, the moisture content of citrus peel samples was monitored until it was below 13% [17], according to the requirement for dried tangerine peel. The final moisture content was determined by oven drying at 105 °C to constant weight. All analyte concentrations were calculated and expressed on a dry-weight basis to eliminate the influence of residual moisture.

Natural air drying (NAD): Citrus peels were spread on a clean grid frame and placed in a ventilated outdoor environment with sufficient light for natural air drying. The ambient temperature was 20–28 °C, the relative humidity was 40–60%, and the drying cycle was 7 d.

Hot air drying (HAD): Citrus peels were spread on a tray and placed in an electric thermostatic blast drying oven. The drying temperature was 60 °C, the wind speed was 1.5 m/s, and the drying cycle was 8 h.

Vacuum drying (VD): Citrus peels were spread on a tray and placed in a vacuum drying oven. The drying temperature was 45 °C, the vacuum degree was −0.09 MPa, and the drying cycle was 10 h.

After drying, the samples were crushed through a 60-mesh sieve, sealed and stored at −20 °C for later use [18].

NOTE: The drying duration and processing parameters were recorded for each treatment. However, drying curves, effective moisture diffusion coefficients, energy consumption, and processing costs were not determined in the present study.

#### 2.2.2. Determination of Physicochemical Indicators

The determination of total flavonoids, total phenols, soluble sugar, and titratable acid content was performed according to the method described by Jiao et al. [19], with appropriate modifications based on the characteristics of citrus peel samples. Total flavonoids were quantified using the aluminum nitrate–sodium nitrite colorimetric method with rutin as the standard. Total phenols were determined using the Folin–Ciocalteu colorimetric method with gallic acid as the standard, while soluble sugars were quantified using the anthrone–sulfuric acid colorimetric method with glucose as the standard. Titratable acid content was measured by acid–base titration using citric acid as the standard. All results were expressed as mg/g dry weight (DW), and each analysis was performed using three independent replicates.

#### 2.2.3. HPLC Analysis of Free Amino Acids

The determination of free amino acids was performed according to the method described by Qian [20] with slight modifications. Briefly, approximately 0.500 g of citrus peel powder was accurately weighed and mixed with 10 mL of ultrapure water. The mixture was vortexed thoroughly and then subjected to ultrasonic extraction at room temperature for 30 min. After cooling to room temperature, the extract was centrifuged at 8000 r/min for 10 min, and the resulting supernatant was filtered through a 0.22 μm aqueous-phase nylon membrane. The filtrate was collected for subsequent derivatization analysis.

For pre-column derivatization, 700 μL of the prepared sample filtrate or 17 amino acid mixed standard solution was mixed with 100 μL of freshly prepared OPA derivatization reagent. The mixture was vortexed thoroughly and subjected to derivatization reaction at room temperature in the dark. After completion of the reaction, the derivatives were immediately analyzed by high-performance liquid chromatography (HPLC).

Chromatographic analysis was performed using a Waters E2695 high-performance liquid chromatography system equipped with a diode array detector (DAD) (Waters Technologies, Milford, MA, USA). Separation was achieved using a Merck C18 column (4.6 mm × 250 mm, 5 μm; Darmstadt, Germany). The column temperature was maintained at 30 °C, with a detection wavelength of 338 nm, flow rate of 1.0 mL/min, and injection volume of 20 μL. A binary high-pressure gradient elution program was applied, and the gradient conditions (Table S1) were optimized to achieve complete baseline separation of the 17 free amino acids. The derivatization reaction was performed using 0.4 mol/L borate buffer (pH 9.5).

#### 2.2.4. GC-MS Analysis of Volatile Flavor Substances

The determination of volatile flavor compounds was performed according to the method described by Xu [21] with slight modifications. Briefly, approximately 0.500 g of citrus peel powder was accurately weighed into a 20 mL headspace vial. Then, 2-methyl-3-heptanone was added as the internal standard for semi-quantitative analysis. Each sample was prepared in triplicate. The vial was sealed and equilibrated in a 60 °C water bath for 30 min. Subsequently, volatile compounds were extracted using a 50/30 μm DVB/CAR/PDMS SPME fiber (Supelco, Bellefonte, PA, USA) at 60 °C for 30 min. After extraction, the fiber was immediately inserted into the GC injection port for desorption at 250 °C for 5 min under splitless mode, and chromatographic acquisition was initiated simultaneously.

Gas chromatography–mass spectrometry (GC–MS) analysis was performed using a Trace TSQ gas chromatography-triple quadrupole mass spectrometer (Thermo Fisher Scientific, Waltham, MA, USA) equipped with a DB-5MS capillary column (30 m × 0.25 mm × 0.25 μm). High-purity helium (≥99.999%) was used as the carrier gas at a constant flow rate of 0.8 mL/min. The injector temperature was maintained at 250 °C under splitless injection mode. The oven temperature program was as follows: the initial temperature was set at 40 °C and maintained for 3 min, increased to 120 °C at 5 °C/min and held for 3 min, increased to 150 °C at 5 °C/min and held for 4 min, and finally increased to 210 °C at 10 °C/min and held for 2 min. The solvent delay time was 3 min.

The mass spectrometer was operated in electron ionization (EI) mode at 70 eV. The ion source temperature and quadrupole temperature were maintained at 230 °C and 150 °C, respectively. Mass spectra were acquired in full-scan mode over an m/z range of 35–450.

Volatile compounds were identified by comparing their mass spectra with those in the National Institute of Standards and Technology (NIST) Chemistry WebBook database. Compounds with a spectral similarity index greater than 80% were considered as tentative identifications. In addition, retention indices (RIs) were calculated using a homologous series of n-alkanes under identical chromatographic conditions and compared with reference values from the NIST database and published literature for further confirmation.

#### 2.2.5. Calculation of Odor Activity Value (OAV)

The aroma contribution ability of volatile compounds depends on their relative concentration, which is quantified by the relative odor threshold (OAV). OAV > 1 was used to determine the key active substances that have actual contribution to the sample aroma [21]. Odor activity values (OAVs) were calculated as the ratio between the concentration of each volatile compound and its corresponding odor threshold. The odor threshold values were obtained from the second edition of Compilation of Odor Threshold Values of Chemical Compounds (Part II). By calculating the OAVs of each volatile compound, their contribution to the overall aroma profile was evaluated, and the formula is as follows:

(1) $$OAV=\frac{C_{i}}{OT_{i}}$$

C_i_ (μg/g) is the concentration of the compound; OT_i_ is the odor threshold of the compound.

#### 2.2.6. Descriptive Sensory Analysis

Descriptive sensory analysis was conducted by a trained panel of 12 members with experience in citrus or tea-like product evaluation. Panelists were trained to assess sweetness, bitterness, sourness, citrus-like aroma, floral aroma, roasted/woody note, aroma intensity, aroma harmony, and overall acceptability. For each sample, 1.00 g of dried citrus peel powder was infused with 50 mL of distilled water at 90 °C for 10 min. The infusion was filtered, cooled to 45 ± 2 °C, coded with random three-digit numbers, and presented in a randomized order. Distilled water was provided for rinsing between samples.

Sensory attributes were scored using a nine-point scale, ranging from 1, extremely weak or unacceptable, to 9, extremely strong or highly acceptable. All evaluations were performed in triplicate. Data were expressed as mean ± standard deviation and ana-lyzed by one-way ANOVA followed by Duncan’s test at *p* < 0.05.

#### 2.2.7. Data Processing and Statistical Analysis

Experimental data were organized using Excel 2021, one-way analysis of variance (ANOVA) was performed using SPSS 26.0 software, and Duncan’s multiple range test was used to test the differences between groups (*p* < 0.05 was considered a significant difference); principal component analysis (PCA) and orthogonal partial least squares discriminant analysis (OPLS-DA) were performed using SIMCA 14.1 software, and 200 permutation tests were performed to verify the model reliability, and key differential compounds with VIP > 1 were screened.

## 3. Results

### 3.1. Effects of Different Drying Methods on the Physicochemical Properties of Citrus Peel

Drying is a critical processing step that influences the retention of bioactive compounds and taste-related substances in citrus peel. To systematically evaluate the effects of different drying techniques on citrus peel quality, four key physicochemical parameters, including total flavonoids, total phenols, soluble sugars, and titratable acids, were determined and compared among the three drying methods (Table 1).

#### 3.1.1. Total Flavonoid Content

Flavonoids are the core bioactive substances of Chenpi, and their content is directly related to the medicinal value and functional characteristics of Chenpi. As shown in Table 1, the total flavonoid content of citrus peel under the three drying methods showed a trend of VD > HAD > NAD (Table 1). Statistical analysis showed that there was no significant difference in total flavonoid content between VD group and HAD group (*p* > 0.05), However, both treatments resulted in significantly higher flavonoid contents than that of the NAD group (*p* < 0.05).

#### 3.1.2. Total Phenol Content

Total phenols constitute an important group of antioxidant compounds in Chenpi and are widely regarded as a key indicator for quality evaluation. The total phenol content was highest in the HAD group, followed by the VD and NAD groups. Nevertheless, no statistically significant differences were detected among the three treatments (*p* > 0.05).

These findings suggest that phenolic compounds exhibit relatively high stability under the drying conditions employed in this study. Although different drying environments were applied, the overall retention of total phenols remained largely unaffected. Therefore, from the perspective of total phenol preservation, all three drying methods appear suitable for maintaining the antioxidant potential of citrus peel.

#### 3.1.3. Soluble Sugar Content

Soluble sugar is the core component of the taste quality of Chenpi, and also participates in the synthesis and transformation of flavor substances, and its content directly affects the sensory coordination of Chenpi. In this study, the soluble sugar content showed significant inter-group differences under the three drying methods (*p* < 0.05), and the differences between the three groups were significant pairwise, specifically showing VD group > NAD group > HAD group (Table 1).

#### 3.1.4. Titratable Acid Content

Titratable acid is the core source of the sour flavor of Chenpi, and also affects the storage stability and flavor coordination of Chenpi. Under the three drying methods, the titratable acid content of citrus peel was in the order of VD group > HAD group = NAD group, and there was no significant statistical difference between groups (*p* > 0.05). Although the titratable acid content in VD group was twice that in HAD group and NAD group, the difference did not reach a significant level due to the intra-group standard deviation of 0, only showing a numerical advantage.

### 3.2. Effects of Different Drying Methods on the Composition and Taste Characteristics of Free Amino Acids in Citrus Peel

Free amino acids (FAAs) are fundamental constituents of proteins and represent important contributors to the taste and flavor quality of citrus peel. In addition to their direct influence on sensory perception, FAAs also serve as key precursors in aroma-forming reactions during food processing [22]. The composition, concentration, and taste-related characteristics of FAAs in citrus peel were markedly influenced by the drying method employed.

#### 3.2.1. Differences in Total Content and Composition of Free Amino Acids

Substantial variations in both the total FAA content and amino acid composition were observed among the three drying treatments. The NAD group exhibited the highest total FAA content, reaching 87.68 mg/g. Isoleucine was the predominant amino acid in this treatment, with a concentration of 29.89 mg/g. In contrast, the HAD group showed the lowest total FAA content (63.41 mg/g), and leucine was identified as the dominant amino acid, accounting for 50.92 mg/g. The total FAA content of the VD group was 74.19 mg/g, with arginine being the most abundant amino acid at 37.56 mg/g.

#### 3.2.2. Differences in Taste Characteristics of Free Amino Acids

In terms of taste characteristics, bitter amino acids dominated under all three treatments, accounting for an average of more than 70% in the three groups of samples, and playing a decisive role in the overall taste profile. Among the three treatments, the HAD group exhibited the highest proportion of bitter amino acids, reaching 86.55%, indicating a pronounced bitter taste characteristic. In comparison, the VD and NAD groups contained relatively higher proportions of sweet amino acids, accounting for 16.62% and 17.74%, respectively. This compositional feature may contribute to improved taste balance and enhanced sensory acceptability.

Umami amino acids were present at relatively low levels in all samples, with an average proportion of only 0.28%, suggesting a limited contribution to the overall flavor profile. Nevertheless, the HAD group contained the highest proportion of umami amino acids (0.49%), corresponding to a concentration of 0.31 mg/g.

Overall, the three drying methods exerted distinct effects on the composition and taste attributes of free amino acids in citrus peel. The most notable differences were observed in bitter amino acids, whose substantial variation among treatments played a decisive role in determining the final taste characteristics of the dried products.

**Figure 1:**
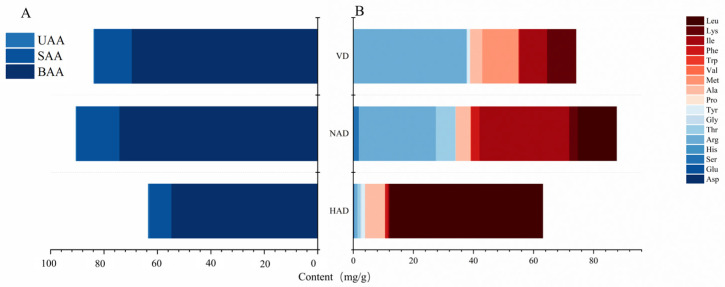
Content proportion of taste-active amino acids of Tangerine Peel under three ways of drying (**A**) Content proportion of total amino acids (**B**).

Among the different taste-related amino acid groups, bitter amino acids exhibited the most pronounced variation among drying treatments (Figure 1A). Arginine showed substantial differences in concentration across samples (Table 2). The HAD group contained only 1.10 mg/g of arginine, whereas markedly higher levels were observed in the NAD and VD groups, reaching 25.66 mg/g and 37.56 mg/g, respectively. In contrast, leucine accumulated predominantly in the HAD treatment, with a concentration of 50.92 mg/g, compared with 12.90 mg/g in the NAD group, while only trace amounts were detected in the VD group. Isoleucine, another major bitter amino acid, also displayed considerable variation among treatments. Its concentration reached 29.89 mg/g in the NAD group but remained relatively low in both the HAD and VD groups. Methionine exhibited comparatively smaller differences among treatments, with the highest concentration detected in the VD group (12.02 mg/g). As major bitter amino acids, methionine, leucine, and isoleucine contribute substantially to the characteristic bitterness of citrus peel and may negatively affect overall taste perception [5].

Distinct differences were also observed in the composition of sweet amino acids. Alanine was most abundant in the HAD group, reaching 6.61 mg/g. The NAD treatment exhibited the greatest diversity of sweet amino acids, with four compounds identified, namely serine, threonine, glycine, and alanine. In comparison, only three sweet amino acids were detected in both the HAD and VD groups. Among these compounds, threonine was particularly enriched in the NAD group, reaching 6.51 mg/g, which was substantially higher than the levels observed under the other drying treatments.

Regarding umami amino acids, aspartic acid and glutamic acid were identified as the primary contributors to umami taste in citrus peel. However, both compounds were present at relatively low concentrations in all samples. Glutamic acid consistently exhibited higher concentrations than aspartic acid across all treatments. The highest glutamic acid content was observed in the HAD group, reaching 0.31 mg/g (Figure 1B). These results suggest that umami amino acids contributed only marginally to the overall taste profile of citrus peel compared with bitter and sweet amino acids.

### 3.3. Effects of Different Drying Methods on Volatile Flavor Substances of Citrus Peel

#### 3.3.1. Differences in Composition and Diversity of Volatile Compounds

**Figure 2:**
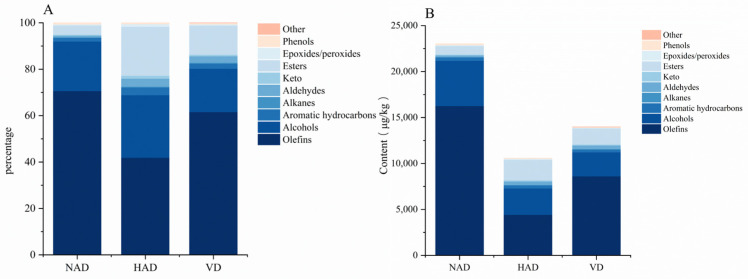
Stacked bar chart of percentage (**A**) and stacked bar chart of content (**B**) of volatile compounds detected by GC-MS.

GC-MS technology was employed to identify and classify volatile compounds in citrus peel subjected to different drying treatments. The results showed that a total of 75 different volatile compounds were detected in the three samples, specifically classified as follows: 32 olefins, 9 aromatic hydrocarbons, 13 alcohols, 6 aldehydes, 6 ketones, 6 esters, 1 phenol and 1 alkane. Horizontal comparative analysis showed that there were certain differences in the species, content and richness of volatile compounds among the three samples, confirming that drying treatment had a significant impact on the composition and content of volatile compounds in citrus peel (Figure 2B).

The number of detected volatile compounds varied among treatments. The VD group exhibited the highest compound diversity, with 70 volatile compounds identified. In contrast, only 64 compounds were detected in the NAD group, suggesting a reduction in volatile diversity. The HAD group contained 66 volatile compounds and therefore exhibited an intermediate level of compound retention (Table 3).

Differences in total volatile content were also observed. The NAD group possessed the highest total concentration of volatile compounds, indicating that natural air drying was particularly effective in preserving volatile substances. Conversely, the HAD group exhibited the lowest total volatile content, suggesting substantial losses during thermal processing. Although the total volatile content of the VD group exceeded that of the HAD group, it remained lower than that observed in the NAD group.

With respect to compound categories, olefins represented the dominant class of volatile compounds in all treatments and constituted the primary source of citrus aroma. The proportion of olefins exceeded 70% in the NAD group (Figure 2A), highlighting their critical contribution to the aroma profile of naturally dried citrus peel. Specifically, the NAD group exhibited the highest concentrations of olefins and alcohols, reaching 16,267.38 ± 3431.58 μg/kg and 4926.21 ± 1988.67 μg/kg, respectively. The volatile profile of the HAD group was characterized by relatively high proportions of olefins, alcohols, and esters. In comparison, the VD treatment displayed a more balanced distribution of volatile compounds, with olefins, alcohols, and esters constituting the major aroma-contributing groups.

#### 3.3.2. Identification of Key Aroma-Active Compounds Based on OAV Analysis

OAV is widely recognized as an effective indicator for evaluating the contribution of individual volatile compounds to overall aroma perception. In the present study, GC–MS data were combined with OAV analysis to identify aroma-active compounds and assess their relative contributions to citrus peel aroma. A total of 33 compounds with OAV values greater than 1 were identified, indicating their direct contribution to aroma perception. These compounds included 10 olefins, 4 alcohols, 6 aldehydes, 1 ketone, and 3 esters (Table 4).

The results in Table 4 indicate that olefin compounds dominated the aroma contribution of citrus peel and were the main skeleton components of citrus peel aroma; however, since aldehydes and alcohols generally have lower olfactory thresholds, they can still present extremely high aroma contribution even at low contents, thus becoming the core differential substances distinguishing the aroma characteristics of the three groups of samples. It is worth noting that linalool, as a representative alcohol compound, had a significantly higher content than other volatile substances and belonged to high aroma contribution substances. Its OAV value ranged from 3164.1 to 3777.97 in the three samples, and its contribution degree to the overall aroma of citrus peel was much higher than that of other compounds, making it the core iconic substance determining the basic aroma characteristics of citrus peel.

From the perspective of characteristic aroma-active substances in different treatment groups, the three groups of samples presented completely different aroma contribution patterns. NAD group had the most abundant types of high aroma contribution active substances. In addition to the core contribution substance linalool, the OAV values of β-myrcene, dodecanal, 1,3,6-octatriene and other substances were significantly higher than those in HAD group and VD group. Among them, 1,3,6-octatriene was only detected in NAD group. These high-activity terpenes and aldehydes further strengthened the terpene fresh fragrance, citrus fragrance and green grass fragrance characteristics of NAD group samples, which was also the core reason for the higher aroma intensity of this group of samples (Table 5).

The aroma profile of the HAD group reflected a pronounced thermal-processing effect. Characteristic aroma-active compounds were mainly represented by α-pinene, 1-cyclohexene-1-carboxaldehyde derivatives, and benzoate-related compounds. These thermally stable terpenes, aldehydes, and esters imparted woody, roasted, and sweet floral notes to the samples. Meanwhile, thermal degradation and volatilization of heat-sensitive terpenes and long-chain aldehydes significantly reduced aroma complexity, resulting in a comparatively simple aroma profile.

In contrast, the VD treatment exhibited the most balanced aroma composition. Key aroma compounds such as linalool were effectively retained, while additional characteristic compounds, including (E)-2-octen-1-ol and benzoate derivatives, were also detected. The low-temperature and reduced-pressure environment minimized the loss of low-boiling-point compounds and alleviated thermal degradation. Consequently, VD generated a well-balanced aroma profile characterized by high richness, complexity, and overall harmony (Table 5).

#### 3.3.3. Multivariate Statistical Analysis of Volatile Compounds

To further clarify the effect of different drying treatments on the composition of volatile compounds in citrus peel, multivariate statistical methods were used for analysis, and the results confirmed that there were significant differences among the three drying treatments.

**Figure 3:**
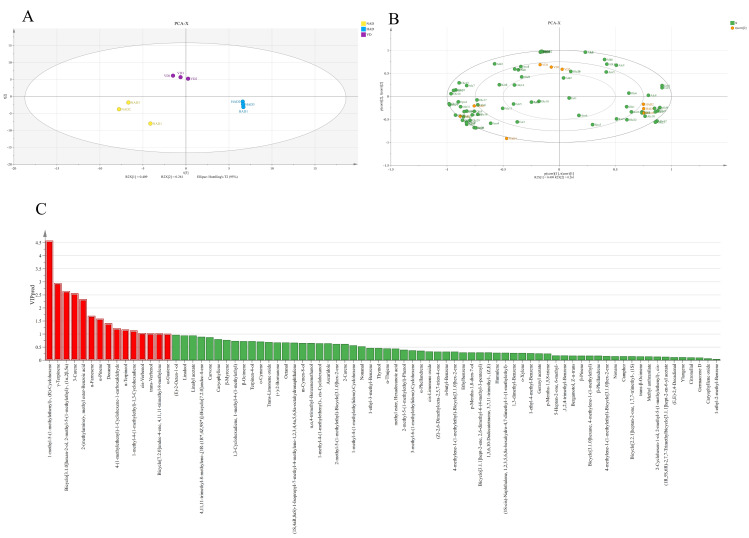
PCA of different drying methods (**A**). PCA of substances under different drying methods (**B**). VIP of all detected volatile substances, red indicates significant substances (VIP > 1) (**C**).

Principal component analysis (PCA) revealed clear separation among samples subjected to different drying treatments. The PCA model explained 67% of the total variance, indicating that the first two principal components effectively captured the major differences in volatile composition among samples. The distinct clustering pattern further confirmed that drying treatment exerted a substantial influence on volatile flavor formation (Figure 3A).

The PCA loading analysis identified the major compounds responsible for sample discrimination. Compounds including nonane, 1,2,4-trimethylbenzene, citronellal, α,α,4-trimethylbenzenemethanol, 3,7,11-trimethyl-1,3,6,10-dodecatetraene, (E)-2-octen-1-ol, and decanal were strongly associated with the VD group and contributed to its rich aroma characteristics. In contrast, compounds such as o-cymene, 3-carene, humulene, 4,11,11-trimethyl-8-methylene-bicyclo[7.2.0]undec-4-ene, 1-methyl-4-(1-methylethylidene)-cyclohexene, and 1-ethyl-2-methylbenzene were predominantly associated with the NAD group. Meanwhile, α-pinene, 2-carene, p-mentha-1,5,8-triene, cis-1-methyl-4-(1-methylethenyl)-cyclohexanol, 6-methyl-5-hepten-2-one, and methyl anthranilate were identified as characteristic compounds of the HAD treatment (Figure 3B).

To further explore the influence mechanism of different drying treatments on the aroma substances of citrus peel, an Orthogonal Partial Least Squares Discriminant Analysis (OPLS-DA) model was constructed, which showed good predictive ability and adaptability. The model evaluation indicators showed that R^2^X = 0.999, R^2^Y = 0.993, Q^2^ = 0.945, all indicators were higher than the threshold of 0.5, confirming that the OPLS-DA model had extremely strong explanatory power and reliable predictability for the differential analysis of volatile compounds in citrus peel (Figure 3A).

**Figure 4:**
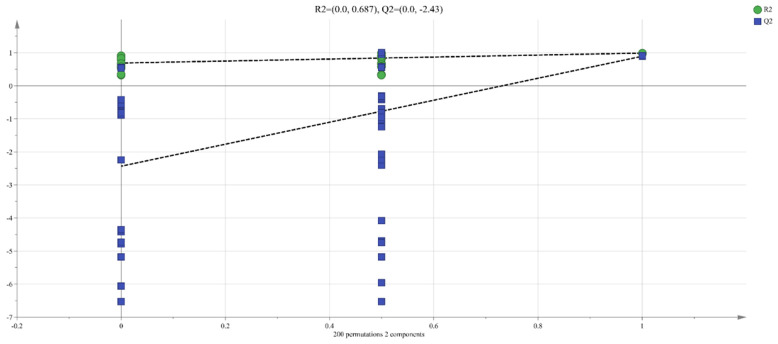
Results of 200 permutation tests.

A 200-permutation test was subsequently conducted to evaluate model reliability and exclude the possibility of overfitting. The results showed that most Q^2^ regression lines intersected below the Y-axis zero point, with specific verification parameters of R^2^ = (0.0, 0.687) and Q^2^ = (0.0, −2.43), further confirming that the model could be used to identify the differences in volatile flavor substances of citrus peel under different drying treatments (Figure 4).

Through Variable Importance in Projection (VIP) analysis, with VIP > 1 as the standard, a total of 16 differential volatile compounds were screened out, which could be used as key markers to distinguish the effects of different drying treatments on volatile compounds in citrus peel (Figure 3C). The category distribution of the 16 key differential compounds was as follows: 8 olefins, 5 alcohols, 2 aldehydes and 1 ester. Among them, the top 10 compounds in terms of VIP value were: (R)-1-methyl-5-(1-methylethenyl)-cyclohexene, γ-terpinene, (1α,2β,5α)-2-methyl-5-(1-methylethyl)-bicyclo[3.1.0]hexan-2-ol, 3-carene, methyl 2-(methylamino)-benzoate, α-farnesene, α-pinene, decanal, 4-(1-methylethenyl)-1-cyclohexene-1-carboxaldehyde, α-terpineol.

#### 3.3.4. Joint Cross Analysis of OAV and VIP

To identify the most critical aroma markers, OAV and VIP analyses were jointly considered. Eight volatile compounds simultaneously satisfying the criteria of OAV > 1 and VIP > 1 were identified, namely α-pinene, γ-terpinene, α-terpineol, decanal, 4-(1-methylethenyl)-1-cyclohexene-1-carboxaldehyde, 3-carene, (E)-2-octen-1-ol, and 1-methyl-4-(1-methylethyl)-1,3-cyclohexadiene.

**Figure 5:**
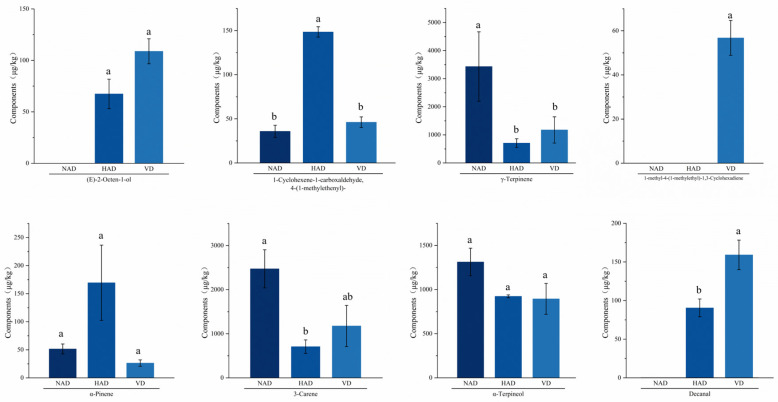
Distribution histogram of key aroma compounds; Different superscript letters in the same row indicate significant differences (*p* < 0.05).

These compounds were regarded as the key differential aroma markers responsible for distinguishing citrus peel processed by different drying methods (Figure 5). The NAD treatment was characterized by the predominance of native terpenes and aldehydes, including γ-terpinene, 3-carene, and decanal, resulting in intense citrus-like and fresh aroma notes [1]. The HAD treatment preferentially retained thermally stable terpenes and oxidation-derived compounds, such as α-terpineol and 4-(1-methylethenyl)-1-cyclohexene-1-carboxaldehyde, generating distinctive roasted and woody aroma characteristics. The VD treatment not only preserved characteristic aroma compounds found in the NAD group but also retained sweet aroma-active compounds such as (E)-2-octen-1-ol. Consequently, VD exhibited a more complex and layered aroma profile that integrated desirable characteristics of both natural air drying and hot-air drying.

#### 3.3.5. Descriptive Sensory Analysis

Sensory evaluation revealed significant differences among the three drying treatments (Table 5). The NAD samples received the highest score for citrus-like aroma and a relatively high score for aroma intensity, indicating that natural air drying was more effective in preserving fresh citrus-like aroma characteristics. However, the overall acceptability of the NAD samples was lower than that of the VD samples, suggesting that aroma intensity alone was not the only factor determining sensory preference.

The HAD samples exhibited the highest scores for bitterness and roasted/woody notes, which may be associated with the higher proportion of bitter amino acids and the stronger thermal-processing characteristics induced by hot-air drying. In contrast, the VD samples obtained the highest scores for sweetness, floral aroma, aroma harmony, and overall acceptability. These findings indicate that vacuum drying produced a more balanced sensory profile, which was generally consistent with its higher soluble sugar content, relatively balanced amino acid composition, and effective retention of key aroma-active compounds.

## 4. Discussion

Drying is a critical step in the conversion of fresh citrus peel into Chenpi and plays a decisive role in determining product quality. Variations in drying conditions, including temperature, oxygen availability, pressure, and light exposure, can substantially influence the retention of bioactive compounds as well as the formation, transformation, and degradation of flavor substances. Consequently, the drying process directly affects the nutritional value and sensory quality of the final product [1,2,20]. This study systematically compared the effects of three common drying methods, NAD, HAD and VD, on the physicochemical properties, free amino acid composition, and volatile flavor substances of *Citrus reticulata* ‘Chachi’ (Xiaoqinggan) peel, revealed the quality regulation mechanism of different drying processes, and provide valuable insights into the quality regulation mechanisms associated with different drying technologies and offer a scientific basis for optimizing Chenpi processing.

Regarding physicochemical properties, VD exhibited the highest retention of total flavonoids and soluble sugars, which is consistent with previous findings reported for fruit and vegetable drying systems [13]. Flavonoids are highly susceptible to oxidation and photodegradation. During NAD, prolonged exposure to light and oxygen may accelerate flavonoid degradation, resulting in lower retention levels. In contrast, both VD and HAD are conducted in enclosed environments that effectively minimize light-induced degradation, thereby improving flavonoid preservation. Similar trends were observed for soluble sugars. Elevated temperatures during HAD may accelerate sugar degradation and promote Maillard reactions, leading to substantial losses of soluble sugars. By comparison, the low-temperature and reduced-pressure conditions employed in VD suppress these reactions and facilitate the retention of soluble sugars [23]. There were no significant differences in total phenols and titratable acid among the three drying methods, indicating that these two types of substances have strong tolerance to changes in drying environment, which is closely related to the material basis that phenolic substances in citrus peel are mostly structurally stable flavonoid polyphenols and titratable acid is dominated by stable organic acids.

Free amino acids are important determinants of taste quality and also serve as key precursors in aroma-forming reactions. Significant differences in amino acid composition and taste characteristics were observed among the three drying methods. The results of this study showed that NAD treatment yielded the highest total free amino acid content and the greatest amino acid diversity, which was because the low-temperature environment of natural air drying reduced the thermal degradation of heat-sensitive amino acids, and the slow drying process provided sufficient time for the enzymatic hydrolysis of proteins, promoting the generation of free amino acids [24,25]. In contrast, the HAD treatment exhibited the lowest total amino acid content but the highest proportion of bitter amino acids, accounting for 86.55% of the total FAA content. Leucine was identified as the predominant amino acid in this treatment. Elevated temperatures may accelerate the degradation of certain heat-sensitive amino acids while simultaneously altering protein hydrolysis pathways, thereby promoting the accumulation of specific bitter amino acids. As a result, a pronounced bitter taste profile was developed. These findings are consistent with previous reports indicating that high-temperature drying often enhances bitterness in fruit- and vegetable-based products [8,26,27]. The amino acid composition of VD group was the most balanced, with a high proportion of sweet amino acids and moderate bitterness, and the retention effect of functional amino acids such as arginine was the best, indicating that vacuum drying can not only guarantee the taste quality, but also better retain the nutritional activity of citrus peel. leucine was not detected in the VD samples under the present analytical conditions, whereas lysine showed a relatively higher level. This result does not necessarily indicate the complete disappearance or formation of specific amino acids during drying. Instead, it may be related to drying-induced changes in matrix composition, extraction efficiency, thermal degradation, amino acid transformation, or pH-dependent solubility and derivatization behavior. Since pH, extraction recovery, and time-dependent amino acid changes were not systematically evaluated in this study, the mechanism underlying these differences remains unclear and requires further investigation.

Volatile compounds are the primary contributors to the characteristic aroma of Chenpi. In this study, 75 volatile compounds were identified, among which olefins constituted the dominant class. This observation agrees with previous reports on the volatile composition of citrus peel and Chenpi products [1]. Terpenoid compounds are widely recognized as the principal source of citrus aroma. The NAD treatment exhibited the highest total concentration of volatile compounds, with olefins accounting for more than 70% of the total volatile content. Consequently, NAD samples displayed the strongest aroma intensity. However, the diversity of volatile compounds was relatively limited, resulting in a comparatively simple aroma profile.

The relatively high volatile content observed in NAD samples may be associated with the slow dehydration process [28]. Gradual moisture removal allows endogenous enzymatic reactions to proceed for a longer period, thereby promoting the generation and accumulation of certain aroma compounds. However, prolonged exposure to oxygen and environmental conditions may simultaneously facilitate the oxidation and degradation of some aroma-active substances, which may explain the relatively lower aroma diversity despite the high overall volatile content.

The HAD treatment showed the lowest total volatile content and was characterized primarily by thermally stable compounds such as α-pinene and ester derivatives. These compounds contributed roasted, woody, and processed aroma notes. Nevertheless, substantial losses of heat-sensitive aroma compounds occurred during thermal drying, reducing overall aroma complexity and richness [4,5,6,7], resulting in insufficient aroma richness.

The inferior aroma retention observed in HAD-treated samples may be attributed not only to thermal degradation but also to drying-induced microstructural changes in citrus peel tissues. Previous studies have shown that hot-air drying can cause cell shrinkage, collapse of cellular structures, and enlargement of pore channels. Such structural damage accelerates mass transfer and facilitates the diffusion and escape of volatile compounds during dehydration. Consequently, aroma-active substances are more readily lost from the tissue matrix, resulting in lower volatile retention and reduced aroma complexity [29].

Moreover, the response of volatile compounds to drying conditions is strongly influenced by their intrinsic physicochemical properties. Heat-sensitive compounds, particularly terpene alcohols and aldehydes with relatively low boiling points, are prone to volatilization, oxidation, and thermal degradation during HAD. In contrast, thermally stable compounds such as α-pinene and certain ester derivatives exhibit greater resistance to heat treatment and therefore become relatively enriched after drying. This selective retention further contributes to the characteristic aroma profile of HAD samples.

In contrast, VD retained the greatest diversity of volatile compounds and exhibited the most balanced aroma composition. Key aroma-active compounds identified by OAV analysis were effectively preserved under vacuum conditions.

The sensory results further supported the chemical composition analysis. The VD samples showed the highest sweetness score, consistent with their higher soluble sugar content, whereas the HAD samples had the highest bitterness score, possibly due to their higher proportion of bitter amino acids. For aroma attributes, the NAD samples received the highest citrus-like aroma score and a relatively high aroma intensity score, which was consistent with their higher volatile compound content. However, their overall acceptability was lower than that of the VD samples, suggesting that aroma intensity alone did not determine sensory preference. Although VD did not show the strongest citrus-like aroma, it obtained the highest scores for floral aroma, aroma harmony, and overall acceptability, indicating a more balanced sensory profile. Overall, the sensory evaluation confirmed that the chemical differences among drying treatments were perceivable by trained panelists, and VD provided a better balance between taste and aroma attributes, supporting its potential advantage in improving the comprehensive flavor quality of dried tangerine peel.

The relatively favorable aroma profile observed in VD may be partially related to its lower drying temperature and reduced oxygen exposure. These conditions could reduce thermal degradation and oxidative loss of heat-sensitive volatile compounds. However, because microstructural analysis was not performed in this study, the possible effects of tissue integrity, structural collapse, and pore formation should be interpreted with caution. In addition, reduced oxygen availability suppresses oxidative degradation, while lower drying temperatures decrease the thermal decomposition of heat-sensitive substances. The combined effects of reduced oxygen exposure and lower processing temperature may contribute to the retention of volatile flavor compounds and bioactive constituents. The role of structural changes requires further confirmation by microstructural observations [30].

The superior aroma quality observed in VD can be attributed to the combined effects of reduced thermal degradation and lower volatilization losses of low-boiling-point compounds under reduced pressure [2], finally forming a rich, coordinated and full aroma system, which was also the core advantage of vacuum drying in flavor quality.

Despite these findings, several limitations should still be acknowledged. First, although sensory evaluation was conducted using a trained panel, consumer acceptance testing was not performed. Therefore, the preference of broader consumer groups requires further investigation. Second, the dynamic changes in physicochemical and flavor-related attributes during drying were not monitored, and drying kinetics, energy consumption, and storage stability were not evaluated. In addition, micro-structural characterization was not performed; therefore, the relationship between tissue structure and volatile retention requires further verification. Future studies should integrate consumer sensory testing, drying kinetics, microstructural analysis, antioxidant activity, and storage experiments to provide a more comprehensive understanding of quality formation during Chenpi processing.

## 5. Conclusions

Drying method significantly affected the nutritional composition and flavor characteristics of *Citrus reticulata* ‘Chachi’ peel. Among the three drying treatments evaluated, VD provided the best overall quality by effectively preserving total flavonoids, soluble sugars, balanced free amino acid composition, and diverse aroma-active compounds. NAD retained the highest total free amino acid and volatile compound contents, whereas HAD resulted in increased bitterness and reduced aroma complexity. Overall, VD achieved the most favorable balance between nutritional quality, taste attributes, and aroma preservation, demonstrating its potential as a preferred drying technology for the production of high-quality Chenpi.

## Figures and Tables

**Table 1:** Physicochemical properties of citrus peels under three drying methods.

Physicochemical Parameters (mg/g)	HAD	NAD	VD
Flavonoids	3.53 ± 0.13 ^a^	2.75 ± 0.38 ^b^	3.77 ± 0.24 ^a^
Total phenols	7.88 ± 1.68 ^a^	6.65 ± 0.07 ^a^	6.94 ± 0.08 ^a^
Soluble sugar	4.20 ± 0.01 ^c^	5.02 ± 0.03 ^b^	5.57 ± 0.00 ^a^
Titratable acid	0.03 ± 0.00 ^a^	0.03 ± 0.00 ^a^	0.06 ± 0.00 ^a^

Note: Data are expressed as mean ± standard deviation. Different superscript letters in the same row indicate significant differences (*p* < 0.05).

**Table 2:** Free amino acid contents of citrus peel samples under three drying methods.

FAA (mg/g)	HAD	NAD	VD
Aspartic	ND	0.02 ± 0.01 ^a^	ND
Glutamic	0.33 ± 0.13 ^a^	0.12 ± 0.05 ^a^	0.16 ± 0.04 ^a^
Serine	ND	2.84 ± 0.57 ^a^	ND
Histidine	ND	ND	ND
Arginine	1.10 ± 0.25 ^b^	25.66 ± 9.01 ^a^	37.56 ± 0.86 ^a^
Threonine	1.30 ± 0.35 ^b^	10.27 ± 1.94 ^a^	0.15 ± 0.09 ^b^
Glycine	0.32 ± 0.02 ^a^	0.08 ± 0.01 ^b^	0.08 ± 0.00 ^b^
Tyrosine	1.24 ± 0.08 ^a^	ND	1.29 ± 0.34 ^a^
Proline	ND	0.07 ± 0.02 ^a^	0.02 ± 0.01 ^b^
Alanine	6.62 ± 0.23 ^a^	5.20 ± 1.33 ^a^	4.05 ± 1.44 ^a^
Methionine	0.06 ± 0.02 ^b^	0.15 ± 0.04 ^b^	16.53 ± 3.78 ^a^
Valine	ND	ND	ND
Tryptophan	ND	0.18 ± 0.03 ^a^	0.05 ± 0.02 ^b^
Phenylalanine	0.93 ± 0.17 ^b^	3.14 ± 0.47 ^a^	0.51 ± 0.11 ^b^
Isoleucine	0.64 ± 0.13 ^b^	29.89 ± 4.6 ^a^	9.13 ± 1.55 ^b^
Lysine	0.30 ± 0.17 ^b^	2.82 ± 1.63 ^ab^	9.63 ± 3.33 ^a^
Leucine	72.18 ± 15.29 ^a^	17.59 ± 4.12 ^b^	ND

Note: Data are expressed as mean ± standard deviation. ND indicates not detected. Different superscript letters in the same row indicate significant differences (*p* < 0.05).

**Table 3:** Contents of volatile substances detected by GC-MS in citrus peel samples under three drying methods.

Compounds (μg/kg)	NAD	HAD	VD
Nonane	14.69 ± 1.93 ^a^	17.89 ± 0.00 ^a^	18.82 ± 0.96 ^a^
1,3-dimethyl-Benzene	9.51 ± 1.20 ^a^	9.02 ± 3.11 ^a^	12.47 ± 0.16 ^a^
Ethylbenzene	9.51 ± 1.20 ^a^	8.39 ± 3.74 ^a^	12.14 ± 0.37 ^a^
o-Xylene	9.51 ± 1.20 ^a^	9.02 ± 3.11 ^a^	12.80 ± 0.37 ^a^
1-ethyl-3-methyl-Benzene	44.26 ± 21.16 ^a^	2.99 ± 0.00 ^a^	3.00 ± 0.01 ^a^
α-Pinene	51.49 ± 8.82 ^a^	169.44 ± 117.03 ^a^	26.31 ± 0.00 ^a^
1,2,4-trimethyl-Benzene	72.33 ± 9.29 ^a^	84.74 ± 0.00 ^a^	82.03 ± 0.19 ^a^
β-Pinene	78.78 ± 10.20 ^a^	70.88 ± 5.48 ^a^	80.00 ± 0.61 ^a^
Bicyclo[3.1.0]hexane, 4-methylene-1-(1-methylethyl)	78.78 ± 10.20 ^a^	76.36 ± 5.48 ^a^	80.00 ± 0.61 ^a^
Octanal	83.92 ± 13.09 ^a^	78.83 ± 16.08 ^a^	96.70 ± 12.14 ^a^
β-Myrcene	206.77 ± 53.23 ^a^	37.83 ± 3.82 ^b^	83.32 ± 19.81 ^b^
2-Carene	121.18 ± 17.48 ^a^	169.70 ± 8.59 ^a^	144.85 ± 23.30 ^a^
(R)-Cyclohexene -1-methyl-5-(1-methylethenyl)	8375.3 ± 1962.18 ^a^	1772.17 ± 476.28 ^b^	3490.86 ± 1399.7 ^ab^
o-Cymene	220.9 ± 79.32 ^a^	204.41 ± 60.31 ^a^	172.47 ± 32.67 ^a^
β-Phellandrene	78.78 ± 10.20 ^a^	73.62 ± 4.75 ^a^	79.70 ± 0.53 ^a^
3-Carene	2472.60 ± 431.88 ^a^	708.02 ± 152.25 ^b^	1176.46 ± 467.99 ^ab^
β-Ocimene	3.11 ± 0.39 ^b^	36.62 ± 0.00 ^a^	2.40 ± 0.53 ^b^
(1α,2β,5α)-Bicyclo[3.1.0]hexan-2-ol, 2-methyl-5-(1-methylethyl)	2020.43 ± 1884.15 ^a^	181.85 ± 27.19 ^a^	119.21 ± 12.73 ^a^
3-methyl-6-(1-methylethylidene)-Cyclohexene	70.75 ± 12.11 ^a^	29.72 ± 0.39 ^b^	45.2 ± 6.06 ^ab^
γ-Terpinene	3433.89 ± 1235.21 ^a^	708.02 ± 152.25 ^a^	1176.46 ± 467.99 ^a^
Nonanal	29.97 ± 5.06 ^ab^	21.06 ± 0.49 ^b^	36.82 ± 2.18 ^a^
Linalool	831.15 ± 132.63 ^a^	738.02 ± 67.07 ^a^	696.11 ± 76.27 ^a^
Camphor	20.38 ± 3.26 ^a^	16.04 ± 1.96 ^a^	19.72 ± 1.88 ^a^
trans-Verbenol	41.39 ± 3.97 ^b^	110.22 ± 0.00 ^a^	40.74 ± 5.35 ^b^
Citronellal	32.2 ± 6.29 ^a^	34.69 ± 4.01 ^a^	36.01 ± 2.93 ^a^
(1S)-Bicyclo[2.2.1]heptan-2-one, 1,7,7-trimethyl	20.38 ± 3.26 ^a^	16.04 ± 1.96 ^a^	19.72 ± 1.88 ^a^
m-Cymen-8-ol	15.08 ± 1.78 ^a^	22.72 ± 0.32 ^a^	37.19 ± 0.00 ^a^
Terpinen-4-ol	570.61 ± 65.18 ^a^	517.65 ± 22.85 ^a^	436.42 ± 76.85 ^a^
α,α,4-trimethyl-Benzenemethanol	15.08 ± 1.78 ^c^	22.91 ± 0.37 ^b^	37.19 ± 0.00 ^a^
α-Terpineol	1312.7 ± 155.78 ^a^	923.5 ± 16.34 ^a^	894.57 ± 174.71 ^a^
Carvone	47.39 ± 4.60 ^b^	84.09 ± 1.11 ^a^	36.52 ± 5.52 ^b^
(1R,5S,6R)-2,7,7-Trimethylbicyclo[3.1.1]hept-2-en-6-yl acetate	12.92 ± 1.49 ^a^	14.24 ± 0.24 ^a^	12.03 ± 2.19 ^a^
Linalyl acetate	831.15 ± 132.63 ^a^	805.09 ± 67.07 ^a^	696.11 ± 76.27 ^a^
Thymol	66.8 ± 2.62 ^a^	17.21 ± 2.30 ^c^	42.42 ± 6.39 ^b^
2-methyl-5-(1-methylethyl)-Phenol	66.8 ± 2.62 ^a^	36.95 ± 2.76 ^b^	42.42 ± 6.39 ^b^
Ylangene	12.62 ± 2.52 ^a^	13.00 ± 1.66 ^a^	10.75 ± 0.02 ^a^
[1R-(1R*,4Z,9S*)]-Bicyclo[7.2.0]undec-4-ene-4,11,11-trimethyl-8-methylene	90.27 ± 50.98 ^a^	52.34 ± 8.94 ^a^	123.95 ± 12.63 ^a^
Caryophyllene	148.6 ± 27.13 ^a^	52.34 ± 8.94 ^b^	123.95 ± 12.63 ^a^
Bicyclo[3.1.1]hept-2-ene, 2,6-dimethyl-6-(4-methyl-3-pentenyl)	24.64 ± 8.58 ^a^	37.94 ± 14.42 ^a^	41.35 ± 10.87 ^a^
Humulene	16.62 ± 2.79 ^a^	4.58 ± 0.97 ^b^	13.24 ± 0.65 ^a^
Bicyclo[7.2.0]undec-4-ene, 4,11,11-trimethyl-8-methylene	148.6 ± 27.13 ^a^	49.01 ± 8.29 ^b^	136.58 ± 0 ^a^
Germacrene D	11.96 ± 1.93 ^a^	8.26 ± 0.63 ^a^	9.26 ± 0.83 ^a^
(Z,E)-1,3,6,10-Dodecatetraene, 3,7,11-trimethyl	24.64 ± 8.58 ^a^	34.61 ± 14.03 ^a^	41.35 ± 10.87 ^a^
Bergamotol, Z-α-trans	10.49 ± 2.02 ^a^	5.49 ± 0.14 ^a^	8.43 ± 1.32 ^a^
methyl ester, Hexadecanoic acid	80.49 ± 53.25 ^a^	12.38 ± 0.00 ^a^	22.56 ± 1.01 ^a^
cis-Verbenol	34.83 ± 0.00 ^b^	100.21 ± 5.96 ^a^	31.46 ± 0.00 ^b^
p-Mentha-1,5,8-triene	6.02 ± 0.00 ^b^	8.3 ± 0.00 ^a^	4.4 ± 0.90 ^b^
cis-Cyclohexanol-1-methyl-4-(1-methylethenyl)	89.51 ± 0.00 ^b^	154.93 ± 27.06 ^a^	119.21 ± 12.73 ^ab^
α-Thujene	69.14 ± 0.00 ^a^	30.61 ± 0.00 ^b^	35.23 ± 5.15 ^b^
1-methyl-4-(1-methylethylidene)-Cyclohexene	114.36 ± 0.00 ^a^	35.8 ± 0.00 ^c^	56.78 ± 7.91 ^b^
α-Farnesene	418.81 ± 0.00 ^a^	76.95 ± 14.82 ^c^	332.04 ± 12.48 ^b^
1-ethyl-2-methyl-Benzene	3.31 ± 0.00 ^a^	2.99 ± 0.00 ^b^	3 ± 0.01 ^b^
5-Hepten-2-one, 6-methyl-	3.13 ± 0.00 ^ab^	4.49 ± 0.95 ^a^	2.4 ± 0.00 ^b^
α-Phellandrene	55.94 ± 0.00 ^a^	30.61 ± 0.00 ^a^	44.15 ± 0.00 ^a^
4-(1-methylethenyl)-1-Cyclohexene-1-carboxaldehyde	35.88 ± 0.00 ^b^	148.44 ± 5.86 ^a^	46.18 ± 5.99 ^b^
1,3-Cyclohexadiene, 1-methyl-4-(1-methylethyl)	90.99 ± 32.20 ^a^	ND	ND
4-methylene-1-(1-methylethyl)-Bicyclo[3.1.0]hex-2-ene	3.70 ± 0.64 ^a^	ND	ND
2-methyl-5-(1-methylethyl)-Bicyclo[3.1.0]hex-2-ene	55.92 ± 7.64 ^a^	ND	ND
trans-β-Ocimene	3.10 ± 0.40 ^a^	ND	ND
(E,E)-2,4-Decadienal	1.97 ± 0.31 ^a^	ND	ND
(E)-2-Octen-1-ol	ND	67.45 ± 14.32 ^b^	108.85 ± 12.14 ^a^
Methyl anthranilate	ND	5.33 ± 0.44 ^a^	3.60 ± 0.27 ^b^
methyl ester-Benzoic acid-2-(methylamino)	ND	1438.27 ± 19.60 ^a^	941.88 ± 148.53 ^b^
Decanal	ND	90.55 ± 11.53 ^b^	159.20 ± 19.09 ^a^
(Z)-2,6-Dimethylocta-2,5,7-trien-4-one	ND	13.57 ± 6.22 ^a^	ND
p-Mentha-1,8-dien-7-ol	ND	7.04 ± 2.19 ^a^	ND
cis-2-Cyclohexen-1-ol, 2-methyl-5-(1-methylethenyl)	ND	1.33 ± 0.08 ^a^	ND
(1S-cis)-Naphthalene, 1,2,3,5,6,8a-hexahydro-4,7-dimethyl-1-(1-methylethyl)-	ND	5.40 ± 0.82 ^a^	ND
1-ethyl-4-methyl-Benzene	ND	ND	2.99 ± 0.01 ^a^
(+)-2-Bornanone	ND	ND	18.63 ± 2.17 ^a^
n-butyl-Benzene	ND	ND	4.43 ± 0.15 ^a^
4-methylene-1-(1-methylethyl)-Bicyclo[3.1.0]hex-2-ene	ND	ND	4.43 ± 0.15 ^a^
1-methyl-4-(1-methylethyl)-1,3-Cyclohexadiene	ND	ND	56.78 ± 7.91 ^a^
Geranyl acetate	ND	ND	2.73 ± 0.23 ^a^
α-Guaiene	ND	ND	47.13 ± 5.6 ^a^
(1S,4aR,8aS)-1-Isopropyl-7-methyl-4-methylene-1,2,3,4,4a,5,6,8a-octahydronaphthalene	ND	ND	18.86 ± 0.58 ^a^

Note: Data are expressed as mean ± standard deviation. ND indicates not detected. Different superscript letters in the same row indicate significant differences (*p* < 0.05).

**Table 4:** OAV values of key compounds in volatile substances of citrus peel samples under three drying methods.

Components	Threshold	NAD	HAD	VD
Octanal	0.8	104.89 ± 16.36 ^a^	88.48 ± 30.16 ^a^	128.47 ± 22.77 ^a^
Nonanal	1.1	27.25 ± 4.60 ^a^	19.30 ± 0.72 ^a^	33.47 ± 3.43 ^a^
α-Pinene	6	10.05 ± 1.47 ^a^	37.99 ± 29.26 ^a^	4.39 ± 0.00 ^a^
β-Phellandrene	36	2.19 ± 0.28 ^a^	2.04 ± 0.23 ^a^	2.21 ± 0.03 ^a^
4-methylene-1-(1-methylethyl)-Bicyclo[3.1.0]hexane	37	2.13 ± 0.28 ^a^	1.99 ± 0.22 ^a^	2.15 ± 0.02 ^a^
β-Myrcene	1.2	216.67 ± 44.36 ^a^	33.12 ± 4.78 ^b^	67.77 ± 28.45 ^ab^
γ-Terpinene	260	13.21 ± 4.75 ^a^	2.43 ± 0.88 ^a^	5.42 ± 2.70 ^a^
Citronellal	6	5.37 ± 1.05 ^a^	5.78 ± 1.16 ^a^	6.00 ± 0.85 ^a^
α-Terpineol	86	15.26 ± 1.81 ^a^	10.74 ± 0.33 ^a^	11.42 ± 3.05 ^a^
Decanal	5.8	35.75 ± 2.34 ^a^	16.61 ± 2.98 ^b^	29.10 ± 4.94 ^ab^
Carvone	27	1.76 ± 0.17 ^b^	3.11 ± 0.07 ^a^	1.45 ± 0.31 ^b^
4-(1-methylethenyl)-1-Cyclohexene-1-carboxaldehyde	30	1.20 ± 0.00 ^b^	4.93 ± 0.34 ^a^	1.44 ± 0.30 ^b^
Linalool	0.22	3777.97 ± 602.85 ^a^	3507.08 ± 457.3 ^a^	3164.11 ± 600.42 ^a^
Dodecanal	0.21	381.82 ± 35.15 ^a^	12.25 ± 3.91 ^b^	27.48 ± 10.92 ^b^
3-Carene	770	3.21 ± 0.56 ^a^	1.12 ± 0.00 ^b^	2.74 ± 0.00 ^ab^
(E)-2-Octen-1-ol	21.4	ND	4.44 ± 2.18 ^a^	5.65 ± 3.95 ^a^
Methyl anthranilate	3	ND	1.50 ± 1.83 ^a^	1.38 ± 1.11 ^a^
2-(methylamino)-methyl ester-Benzoic acid	349	ND	4.01 ± 4.18 ^a^	3.55 ± 2.27 ^a^
1-methyl-4-(1-methylethenyl)-, (S)-Cyclohexene	1040	ND	1.25 ± 0.00 ^a^	ND
Undecanal	12.5	6.41 ± 1.02 ^a^	ND	1.61 ± 0.00 ^a^
Caryophyllene	64	2.32 ± 0.73 ^a^	ND	2.13 ± 1.54 ^a^
(E,E)-2,4-Decadienal	0.077	27.59 ± 8.53 ^a^	ND	ND
2,4-Decadienal	0.3	8.63 ± 0.00 ^a^	ND	ND
(Z)-3,7-dimethyl-1,3,6-Octatriene	34	529.00 ± 0.00 ^a^	ND	ND
1-methyl-4-(1-methylethyl)-1,3-Cyclohexadiene	80	1.64 ± 0.41 ^a^	ND	ND
Terpinen-4-ol	590	1.08 ± 0.04 ^a^	ND	1.00 ± 0.00 ^a^
α-Phellandrene	40	1.40 ± 0.00 ^a^	ND	ND
Linalyl acetate	1000	1.07 ± 0.00 ^a^	ND	ND
Phenol, 2-methyl-5-(1-methylethyl)-Phenol	70	1.00 ± 0.00 ^a^	ND	ND
1-methyl-4-(1-methylethylidene)-Cyclohexene	200	ND	ND	1.72 ± 1.05 ^a^
1-(4-methylphenyl)-Ethanone	21	ND	ND	1.10 ± 0.00 ^a^
Limonene	200	3.98 ± 0.00 ^a^	13.62 ± 0.00 ^a^	ND
6,6-dimethyl-Bicyclo[3.1.1]hept-2-ene-2-methanol	7	1.52 ± 0.00 ^a^	1.48 ± 0.00 ^a^	ND

Note: Data are expressed as mean ± standard deviation. ND indicates not detected. Different superscript letters in the same row indicate significant differences (*p* < 0.05).

**Table 5:** Descriptive sensory analysis of dried tangerine peel infusions prepared by different drying methods.

Sensory Attribute	NAD	HAD	VD
Sweetness	5.43 ± 0.58 ^b^	4.38 ± 0.49 ^c^	6.21 ± 0.52 ^a^
Bitterness	4.96 ± 0.61 ^b^	6.42 ± 0.58 ^a^	4.51 ± 0.54 ^b^
Sourness	4.36 ± 0.43 ^b^	4.18 ± 0.47 ^b^	4.87 ± 0.51 ^a^
Citrus-like aroma	7.18 ± 0.55 ^a^	5.52 ± 0.63 ^c^	6.76 ± 0.49 ^b^
Floral aroma	5.74 ± 0.48 ^b^	4.91 ± 0.52 ^c^	6.33 ± 0.46 ^a^
Green/fresh note	6.48 ± 0.59 ^a^	4.67 ± 0.55 ^c^	5.86 ± 0.53 ^b^
Roasted/woody note	4.12 ± 0.50 ^c^	6.18 ± 0.62 ^a^	4.68 ± 0.47 ^b^
Aroma intensity	7.05 ± 0.57 ^a^	5.74 ± 0.61 ^b^	6.83 ± 0.54 ^a^
Aroma harmony	6.08 ± 0.52 ^b^	5.03 ± 0.56 ^c^	7.12 ± 0.48 ^a^
Overall acceptability	6.26 ± 0.55 ^b^	5.17 ± 0.53 ^c^	7.28 ± 0.50 ^a^

Note: Data are expressed as mean ± standard deviation. Different lowercase letters in the same row indicate significant differences among treatments at *p* < 0.05.

## Data Availability

All related data and methods are presented in this paper. Additional inquiries should be addressed to the corresponding author.

## Supplementary Materials

The following supporting information can be downloaded at: https://www.mdpi.com/article/10.3390/foods15152669/s1, Table S1 Scheme of elution gradient for HPLC-DAD analysis. Table S2 The composition and content of VOCs in peels of *Citrus reticulata* with drying Methods. Table S3 Identification and quantification information of volatile compounds detected in citrus peel samples by SPME-GC-MS.

## References

[1] Wang Z.R., Tao Z., Mei X.F., Liu Y. (2023). Comparison of different drying technologies for brocade orange peels: Changes in color, phytochemical properties, volatile profile, bioaccessibility and bioactive compounds. Food Chem..

[2] Ma J., Wang Y., Li J., Zhang J., Wei Y., Yan Y., Wang H., Pan Y., Xiong Z., Wang R. (2024). Aroma formation mechanism by the drying step during Congou black tea processing: Analyses by HP-SPME and SAFE with GC-MS. LWT—Food Sci. Technol..

[3] Zhai X.T., Zhang L., Granvogl M., Ho C.T., Wan X.C. (2022). Flavor of tea: A review on odorants and analytical techniques. Compr. Rev. Food Sci. Food Saf..

[4] Feng Z.H., Li Y.F., Li M., Wang Y.J., Zhang L., Wan X. (2019). Tea aroma formation from six model manufacturing processes. Food Chem..

[5] Liu N.F., Shen S.S., Huang L.F., Deng G.J., Wei Y.M., Ning J.M. (2023). Revelation of volatile contributions in green teas with different aroma types by GC-MS and GC-IMS. Food Res. Int..

[6] Chen Q.C., Zhu Y., Dai W.D., Lv H.P., Mu B., Li P.L. (2019). Aroma formation and dynamic changes during white tea processing. Food Chem..

[7] Wei Y.M., Yin X.C., Wu H.T., Zhao M.J., Huang J.L., Zhang J.X. (2022). Improving the flavor of summer green tea using the yellowing process. Food Chem..

[8] Wang C., Zhang C.X., Kong Y.W., Peng X.P., Li C.W., Liu S.H. (2017). A comparative study of volatile components in Dianhong teas from fresh leaves of four tea cultivars by using chromatography-mass spectrometry, multivariate data analysis, and descriptive sensory analysis. Food Res. Int..

[9] Kang S.Y., Yan H., Zhu Y., Liu X., Lv H.P., Zhang Y., Ho C.T. (2019). Identification and quantification of key odorants in the world’s four most famous black teas. Food Res. Int..

[10] Kinoshita T., Hirata S., Yang Z.Y., Baldermann S., Kitayama E., Matsumoto S., Watanabe N. (2010). Formation of damascenone derived from glycosidically bound precursors in green tea infusions. Food Chem..

[11] Zeng L.T., Zhou Y., Gui J.D., Fu X.M., Mei X., Zhen Y.P. (2016). Formation of volatile tea constituent indole during the oolong tea manufacturing process. J. Agric. Food Chem..

[12] Bahorun T., Luximon-Ramma A., Gunness T., Sookar D., Aruoma O.I. (2010). Black tea reduces uric acid and C-reactive protein levels in humans susceptible to cardiovascular diseases. Toxicology.

[13] Wu F., Tang J., Pei F., Zhou C. (2015). The influence of four drying methods on nonvolatile taste components of White *Hypsizygus marmoreus*. Eur. Food Res. Technol..

[14] Zhang L., Ho C.T., Zhou J., Santos J.S., Armstrong L., Granato D. (2019). Chemistry and biological activities of processed *Camellia sinensis* teas: A comprehensive review. Compr. Rev. Food Sci. Food Saf..

[15] Jumtee K., Komura H., Bamba T., Fukusaki E. (2011). Prediction of Japanese green tea (Sen-cha) ranking by volatile profiling using gas chromatography mass spectrometry and multivariate analysis. J. Biosci. Bioeng..

[16] Xiao Z.B., Wang H.L., Niu Y.W., Liu Q., Zhu J.C., Chen H.X. (2017). Characterization of aroma compositions in different Chinese congou black teas using GC-MS and GC-O combined with partial least squares regression. Flavour Fragr. J..

[17] (2013). Tea Storage; General Administration of Quality Supervision, Inspection and Quarantine of the People’s Republic of China.

[18] Huang W.J., Fang S.M., Wang J., Zhuo C., Luo Y.H., Yu Y.L. (2022). Sensomics analysis of the effect of the withering method on the aroma components of Keemun black tea. Food Chem..

[19] Xie J., Deng B., Wang W., Zhang H. (2023). Changes in Sugar, Organic Acid and Free Amino Acid Levels and the Expression of Genes Involved in the Primary Metabolism of Oleocellosis in Citrus Peels. J. Plant Physiol..

[20] Qian C.L., Jiang Y.Y., Sun Y., Chen J. (2023). Changes in the texture and flavor of lotus root after different cooking methods. Foods.

[21] Xu M., Wang F., Gu Y., Zhang B., Shao Y., Liu J., Kan J., Zhang M., Xiao L., Qi X. (2026). GC–IMS and GC–MS Combined With Multivariate Analysis for Objective Evaluation of Flavor Quality Differences Among Lotus Root Varieties. J. Food Sci..

[22] Yang Z.Y., Baldermann S., Watanabe N. (2013). Recent studies of the volatile compounds in tea. Food Res. Int..

[23] Zhang J., Chen J., Lan J., Li Y. (2024). Effect of different drying techniques on the bioactive compounds, antioxidant ability, sensory and volatile flavor compounds of mulberry. Foods.

[24] Li J.Y., Hao C.H., Jia H.Y., Zhang J., Wu H.T., Ning J.M. (2022). Aroma characterization and their changes during the processing of black teas from the cultivar, *Camellia sinensis* (L.) O. Kuntze cv. Jinmudan. J. Food Compos. Anal..

[25] Sun M.X., Ni L.Y., Huang Y.H., Yang M.L., Cheng G.G., Zhang M., Wu M.Y., Ma C. (2023). Effects of different drying treatments on the microstructure, free amino acids, volatile compounds and antioxidant activity of *Flammulina velutipes* root. Food Chem. X.

[26] Zhu J.C., Niu Y.W., Xiao Z.B. (2020). Characterization of the key aroma compounds in Laoshan green teas by application of odour activity value (OAV), gas chromatography-mass spectrometry-olfactometry (GC-MS-O) and comprehensive two-dimensional gas chromatography mass spectrometry (GC × GC-qMS). Food Chem..

[27] Guo X.Y., Ho C.T., Schwab W., Wan X.C. (2021). Aroma profiles of green tea made with fresh tea leaves plucked in summer. Food Chem..

[28] Ge S., Chen Y., Ding S., Zhou H., Jiang L., Yi Y., Deng F., Wang R. (2020). Changes in volatile flavor compounds of peppers during hot air drying process based on headspace-gas chromatography-ion mobility spectrometry (HS-GC-IMS). J. Sci. Food Agric..

[29] Tu D., Xiao H., Li L., Xu Y., Cheng L., Zhao Y., Lin Y., Xiang Q., Tian Y. (2026). Impact of airborne ultrasound on pore characteristics and cell wall components in shiitake mushrooms during microwave vacuum drying. Food Chem. X.

[30] Pachura N., Gavahian M., Jałoszyński K., Surma M., Szumny A. (2025). Changes in bioactive and aroma compounds in flower elderberry as affected by innovative drying method. Ind. Crops Prod..

