# Supplementary material for: Effects of Drying Methods on Flavor Substances and Identification of Characteristic Flavor Compounds of Dried Tangerine Peel
Source: Foods. 2026 Jul 29;15(15):2669. doi: 10.3390/foods15152669 (PMC13464984; doi:10.3390/foods15152669)
Supplement: Supplementary file 1 [file foods-15-02669-s001.zip › foods-4409727-supplementary.pdf]

Supplementary material

Table S1 Scheme of elution gradient for HPLC-DAD analysis.

Time (min)	Solvent (A %)	Solvent (B %)
0	10	90
10	18	82
15	24	76
21	41	59
21.5	41	59
22	42.2	57.8
23	42.5	57.5
27	58	42
29	59	41
31	59.5	40.5
32	60	40
38	60	40
40	10	90
45	10	90

Table S2 The composition and content of VOCs in peels of *Citrus reticulata* with drying Methods

	VOCs（μg/kg）	NAD	HAD	VD
Alc1	Linalool	831.15±132.63a	738.02±67.07a	696.11±76.27a
Alc2	cis-Verbenol	34.83±0.00b	100.21±5.96a	31.46±0.00b
Alc3	trans-Verbenol	41.39±3.97b	110.22±0.00a	40.74±5.35b
Alc4	m-Cymen-8-ol	15.08±1.78a	22.72±0.32a	37.19±0.00a
Alc5	Terpinen-4-ol	570.61±65.18a	517.65±22.85a	436.42±76.85a
Alc6	α-Terpineol	1312.70±155.78a	923.50±16.34a	894.57±174.71a
Alc7	Bergamotol, Z-α-trans	10.49±2.02a	5.49±0.14a	8.43±1.32a
Alc8	Cyclohexanol, 1-methyl-4-(1-methylethenyl)-, cis-	89.51±0.00b	154.93±27.06a	119.21±12.73ab

Alc9	2-Octen-1-ol, (E)-	ND	67.45±14.32b	108.85±12.14a
Alc10	p-Mentha-1,8-dien-7-ol	ND	7.04±2.19a	ND
Alc11	Bicyclo[3.1.0]hexan-2-ol, 2-methyl-5-(1-methylethyl)-, (1α,2β,5α)	2020.43±1884.15a	181.85±27.19a	119.21±12.73a
Alc12	2-Cyclohexen-1-ol, 2-methyl-5-(1-methylethenyl)-, cis-	ND	1.33±0.08a	ND
Ole1	α-Pinene	51.49±8.82a	169.44±117.03a	26.31±0.00a
Ole2	β-Pinene	78.78±10.20a	70.88±5.48a	80.00±0.61a
Ole3	Bicyclo[3.1.0]hexane, 4-methylene-1-(1-methylethyl)	78.78±10.20a	76.36±5.48a	80.00±0.61a
Ole4	β-Myrcene	206.77±53.23a	37.83±3.82b	83.32±19.81b
Ole5	β-Phellandrene	78.78±10.20a	73.62±4.75a	79.7±0.53a
Ole6	2-Carene	121.18±17.48a	169.7±8.59a	144.85±23.30a
Ole7	3-Carene	2472.6±431.88a	708.02±152.25b	1176.46±467.99ab
Ole8	β-Ocimene	3.11±0.39b	36.62±0.00a	2.40±0.53b
Ole9	γ-Terpinene	3433.89±1235.21a	708.02±152.25a	1176.46±467.99a
Ole10	Ylangene	12.62±2.52a	13±1.66a	10.75±0.02a
Ole11	Caryophyllene	148.6±27.13a	52.34±8.94b	123.95±12.63a
Ole12	Humulene	16.62±2.79a	4.58±0.97b	13.24±0.65a
Ole13	Bicyclo[3.1.1]hept-2-ene, 2,6-dimethyl-6-(4-methyl-3-pentenyl)	24.64±8.58a	37.94±14.42a	41.35±10.87a
Ole14	Bicyclo[7.2.0]undec-4-ene, 4,11,11-trimethyl-8-methylene-,[1R-(1R*,4Z,9S*)]	90.27±50.98a	52.34±8.94a	123.95±12.63a
Ole15	Bicyclo[7.2.0]undec-4-ene, 4,11,11-trimethyl-8-methylene	148.60±27.13a	49.01±8.29b	136.58±0.00a
Ole16	α-Farnesene	418.81±0.00a	76.95±14.82c	332.04±12.48b
Ole17	Germacrene D	11.96±1.93a	8.26±0.63a	9.26±0.83a
Ole18	1,3-Cyclohexadiene, 1-methyl-4-(1-methylethyl)	90.99±32.2a	ND	ND
Ole19	1,3-Cyclohexadiene, 1-methyl-4-(1-methylethyl)-	ND	ND	56.78±7.91a

Ole20	1,3,6,10-Dodecatetraene, 3,7,11-trimethyl-, (Z,E)	24.64±8.58a	34.61±14.03a	41.35±10.87a
Ole21	Cyclohexene, 1-methyl-5-(1-methylethenyl)-, (R)-	8375.30±1962.18a	1772.17±476.28b	3490.86±1399.70ab
Ole22	p-Mentha-1,5,8-triene	6.02±0.00b	8.30±0.00a	4.4±0.90b
Ole23	α-Thujene	69.14±0.00a	30.61±0.00b	35.23±5.15b
Ole24	α-Guaiene	ND	ND	47.13±5.60a
Ole25	Naphthalene, 1,2,3,5,6,8a-hexahydro-4,7-dimethyl-1-(1-methylethyl)-, (1S-cis)-	ND	5.4±0.82a	ND
Ole26	Bicyclo[3.1.0]hex-2-ene, 2-methyl-5-(1-methylethyl)-	55.92±7.64a	ND	ND
Ole27	Cyclohexene, 1-methyl-4-(1-methylethylidene)	114.36±0.00a	35.8±0.00c	56.78±7.91b
Ole28	α-Phellandrene	55.94±0.00a	30.61±0.00a	44.15±0.00a
Ole29	Bicyclo[3.1.0]hex-2-ene, 4-methylene-1-(1-methylethyl)	3.70±0.64a	ND	ND
Ole30	trans-β-Ocimene	3.10±0.4a	ND	ND
Ole31	Cyclohexene, 3-methyl-6-(1-methylethylidene)	70.75±12.11a	29.72±0.39b	45.2±6.06ab
Ole32	(1S,4aR,8aS)-1-Isopropyl-7-methyl-4-methylene-1,2,3,4,4a,5,6,8a-octahydronaphthalene	ND	ND	18.86±0.58a
Alk1	Nonane	14.69±1.93a	17.89±0.00a	18.82±0.96a
Alk2	Benzenemethanol, α,α,4-trimethyl	15.08±1.78c	22.91±0.37b	37.19±0.00a
Aro1	Benzene, 1,3-dimethyl-	9.51±1.20a	9.02±3.11a	12.47±0.16a
Aro2	Ethylbenzene	9.51±1.20a	8.39±3.74a	12.14±0.37a
Aro3	o-Xylene	9.51±1.20a	9.02±3.11a	12.8±0.37a
Aro4	Benzene, 1-ethyl-3-methyl-	44.26±21.16a	2.99±0.00a	3.00±0.01a
Aro5	Benzene, 1,2,4-trimethyl	72.33±9.29a	84.74±0.00a	82.03±0.19a
Aro6	o-Cymene	220.9±79.32a	204.41±60.31a	172.47±32.67a
Aro7	Benzene, 1-ethyl-2-methyl-	3.31±0.00a	2.99±0.00b	3.00±0.01b
Aro8	Benzene, n-butyl-	ND	ND	4.43±0.15a
Est1	(1R,5S,6R)-2,7,7-Trimethylbicyclo[3.1.1]hept-	12.92±1.49a	14.24±0.24a	12.03±2.19a

	2-en-6-yl acetate			
Est2	Linalyl acetate	831.15±132.63a	805.09±67.07a	696.11±76.27a
Est3	Hexadecanoic acid, methyl ester	80.49±53.25a	12.38±0.00a	22.56±1.01a
Est4	Methyl anthranilate	ND	5.33±0.44a	3.60±0.27b
Est5	Benzoic acid, 2-(methylamino)-, methyl ester	ND	1438.27±19.60a	941.88±148.53b
Est6	Geranyl acetate	ND	ND	2.73±0.23a
Ald1	Octanal	83.92±13.09a	78.83±16.08a	96.7±12.14a
Ald3	Nonanal	29.97±5.06ab	21.06±0.49b	36.82±2.18a
Ald2	Citronellal	32.20±6.29a	34.69±4.01a	36.01±2.93a
Ald4	1-Cyclohexene-1-carboxaldehyde, 4-(1-methylethenyl)-	35.88±0.00b	148.44±5.86a	46.18±5.99b
Ald5	2,4-Decadienal, (E,E)-	1.97±0.31a	ND	ND
Ald6	Decanal	ND	90.55±11.53b	159.2±19.09a
Ket1	Camphor	20.38±3.26a	16.04±1.96a	19.72±1.88a
Ket2	Bicyclo[2.2.1]heptan-2-one, 1,7,7-trimethyl-, (1S)	20.38±3.26a	16.04±1.96a	19.72±1.88a
Ket4	Carvone	47.39±4.60b	84.09±1.11a	36.52±5.52b
Ket5	5-Hepten-2-one, 6-methyl-	3.13±0.00ab	4.49±0.95a	2.40±0.00b
Ket6	(Z)-2,6-Dimethylocta-2,5,7-trien-4-one	ND	13.57±6.22a	ND
Ket3	(+)-2-Bornanone	ND	ND	18.63±2.17a
Phe1	Thymol	66.8±2.62a	17.21±2.30c	42.42±6.39b
Phe2	Phenol, 2-methyl-5-(1-methylethyl)	66.8±2.62a	36.95±2.76b	42.42±6.39b
Oth2	Benzene, 1-ethyl-4-methyl-	ND	ND	2.99±0.01a
Oth1	Bicyclo[3.1.0]hex-2-ene, 4-methylene-1-(1-methylethyl)-	ND	ND	4.43±0.15a
Epo1	Limonene oxide, trans	18.68±3.63b	44.60±5.75a	13.71±1.22b
Epo3	Ascaridole	67.79±0.00a	75.48±1.98a	59.01±8.81a
Epo4	Caryophyllene oxide	2.14±0.00a	1.20±0.27b	1.55±0.12ab
Epo2	Limonene oxide, cis	15.84±1.26a	ND	ND

Note: Data are expressed as mean ± standard deviation. ND indicates not detected. different superscript letters in the same row indicate significant differences ($P < 0.05$).

Table S3 Identification and quantification information of volatile compounds detected in citrus peel samples by SPME-GC-MS

	CAS	Compounds	RT (min)	RI(ref)	SI (%)	NAD	HAD	VD
Alc1	78-70-6	Linalool	14.84	1100	94.3	831.15±132.63a	738.02±67.07a	696.11±76.27a
Alc2	1845-30-3	cis-Verbenol	15.53	1124	82.3	34.83±0.00b	100.21±5.96a	31.46±0.00b
Alc3	1820-09-3	trans-Verbenol	15.53	1131	81.8	41.39±3.97b	110.22±0.00a	40.74±5.35b
Alc4	5208-37-7	m-Cymen-8-ol	17.68	1155	82.9	15.08±1.78a	22.72±0.32a	37.19±0.00a
Alc5	562-74-3	Terpinen-4-ol	17.44	1172	85.8	570.61±65.18a	517.65±22.85a	436.42±76.85a
Alc6	98-55-5	α-Terpineol	17.88	1182	91.7	1312.7±155.78a	923.5±16.34a	894.57±174.71a
Alc8	7299-41-4	Cyclohexanol, 1-methyl-4-(1-methylethenyl)-, cis-	13.65	1129	92.8	89.51±0.00b	154.93±27.06a	119.21±12.73ab
Alc11	15537-55-0	Bicyclo[3.1.0]hexan-2-ol, 2-methyl-5-(1-methylethyl)-, (1α,2β,5α)	13.65	1054	89.4	2020.43±1884.15a	181.85±27.19a	119.21±12.73a
Ole1	80-56-8	α-Pinene	8.26	942	87.7	51.49±8.82a	169.44±117.03a	26.31±0.00a
Ole2	127-91-3	β-Pinene	10.1	964	88.8	78.78±10.20a	70.88±5.48a	80.00±0.61a
Ole3	3387-41-5	Bicyclo[3.1.0]hexane, 4-methylene-1-(1-methylethyl)	10.1	974	91.4	78.78±10.20a	76.36±5.48a	80.00±0.61a
Ole4	123-35-3	β-Myrcene	10.82	988	90	206.77±53.23a	37.83±3.82b	83.32±19.81b
Ole5	555-10-2	β-Phellandrene	10.1	1011	86	78.78±10.20a	73.62±4.75a	79.70±0.53a
Ole6	554-61-0	2-Carene	19.35	995	85	121.18±17.48a	169.7±8.59a	144.85±23.30a
Ole7	13466-78-9	3-Carene	13.38	1015	88.5	2472.60±431.88a	708.02±152.25b	1176.46±467.99ab
Ole8	13877-	β-Ocimene	8.52	1043	86.6	3.11±0.39b	36.62±0.00a	2.40±0.53b

	91-3							
Ole9	99-85-4	γ-Terpinene	13.38	1079	90	3433.89±1235.21a	708.02±152.25a	1176.46±467.99a
Ole10	14912-44-8	Ylangene	23.38	1351	85.2	12.62±2.52a	13±1.66a	10.75±0.02a
Ole11	87-44-5	Caryophyllene	24.57	1429	90.2	148.60±27.13a	52.34±8.94b	123.95±12.63a
Ole12	6753-98-6	Humulene	25.47	1455	87.1	16.62±2.79a	4.58±0.97b	13.24±0.65a
Ole14	118-65-0	Bicyclo[7.2.0]undec-4-ene, 4,11,11-trimethyl-8-methylene-,[1R-(1R*,4Z,9S*)]	24.57	1403	89.1	90.27±50.98a	52.34±8.94a	123.95±12.63a
Ole15	13877-93-5	Bicyclo[7.2.0]undec-4-ene, 4,11,11-trimethyl-8-methylene	24.57	1476	85.4	148.6±27.13a	49.01±8.29b	136.58±0.00a
Ole16	502-61-4	α-Farnesene	26.96	1490	83.9	418.81±0.00a	76.95±14.82c	332.04±12.48b
Ole18	99-86-5	1,3-Cyclohexadiene, 1-methyl-4-(1-methylethyl)	11.77	1020	91	90.99±32.2a	ND	ND
Ole18	99-86-5	1,3-Cyclohexadiene, 1-methyl-4-(1-methylethyl)-	11.77	1020	91	90.99±32.2a	ND	ND
Ole21	1461-27-4	Cyclohexene, 1-methyl-5-(1-methylethenyl)-, (R)-	12.25	1006	89.7	8375.3±1962.18a	1772.17±476.28b	3490.86±1399.7ab
Ole22	21195-59-5	p-Mentha-1,5,8-triene	15.25	1112	84.8	6.02±0.00b	8.30±0.00a	4.40±0.9b
Ole23	2867-05-2	α-Thujene	8.26	934	91.4	69.14±0.00a	30.61±0.00b	35.23±5.15b
Ole24	3691-12-1	α-Guaiene	26.68	1428	83.7	ND	ND	47.13±5.60a
Ole25	483-76-1	Naphthalene, 1,2,3,5,6,8a-hexahydro-4,7-dimethyl-1-(1-methylethyl)-, (1S-cis)-	27.55	1519	80.5	ND	5.40±0.82a	ND
Ole26	2867-05-2	Bicyclo[3.1.0]hex-2-ene, 2-methyl-5-(1-methylethyl)-	8.26	934	91.4	55.92±7.64a	ND	ND
Ole27	586-62-9	Cyclohexene, 1-methyl-4-(1-methylethylidene)	11.77	1084	89.7	114.36±0.00a	35.80±0.00c	56.78±7.91b

Ole28	99-83-2	α-Phellandrene	8.26	1003	88.1	55.94±0.00a	30.61±0.00a	44.15±0.00a
Ole30	3779-61-1	trans-β-Ocimene	12.99	1059	87.6	3.10±0.40a	ND	ND
Ole31	586-63-0	Cyclohexene, 3-methyl-6-(1-methylethylidene)	14.44	1057	89.9	70.75±12.11a	29.72±0.39b	45.2±6.06ab
Ole32	6980-46-7	(1S,4aR,8aS)-1-Isopropyl-7-methyl-4-methylene-1,2,3,4,4a,5,6,8a-octahydronaphthalene	27.55	1483	83.7	ND	ND	18.86±0.58a
Alk1	111-84-2	Nonane	7.2	138.27	88.8	14.69±1.93a	17.89±0.00a	18.82±0.96a
Aro1	108-38-3	Benzene, 1,3-dimethyl-	6.24	845	88.3	9.51±1.20a	9.02±3.11a	12.47±0.16a
Aro2	100-41-4	Ethylbenzene	6.03	846	94.4	9.51±1.20a	8.39±3.74a	12.14±0.37a
Aro3	95-47-6	o-Xylene	6.24	879	87.5	9.51±1.20a	9.02±3.11a	12.8±0.37a
Aro4	620-14-4	Benzene, 1-ethyl-3-methyl-	9.62	939	91.8	44.26±21.16a	2.99±0.00a	3.00±0.01a
Aro5	95-63-6	Benzene, 1,2,4-trimethyl	9.89	956	93.4	72.33±9.29a	84.74±0.00a	82.03±0.19a
Aro6	527-84-4	o-Cymene	12.07	1009	94.5	220.9±79.32a	204.41±60.31a	172.47±32.67a
Aro7	611-14-3	Benzene, 1-ethyl-2-methyl-	9.62	955	90.1	3.31±0.00a	2.99±0.00b	3.00±0.01b
Aro8	104-51-8	Benzene, n-butyl-	8.95	1046.3	80.3	ND	ND	4.43±0.15a
Est2	115-95-7	Linalyl acetate	14.84	1251	82	831.15±132.63a	805.09±67.07a	696.11±76.27a
Est3	112-39-0	Hexadecanoic acid, methyl ester	36.28	1910	88.4	80.49±53.25a	12.38±0.00a	22.56±1.01a
Est4	134-20-3	Methyl anthranilate	22.33	1332	84.3	ND	5.33±0.44a	3.6±0.27b

Est5	85-91-6	Benzoic acid, 2-(methylamino)-, methyl ester	24.18	1375	91.3	ND	1438.27±19.60a	941.88±148.53b
Est6	105-87-3	Geranyl acetate	23.47	1363	81.7	ND	ND	2.73±0.23a
Ald3	124-19-6	Nonanal	14.98	1083	82.4	29.97±5.06ab	21.06±0.49b	36.82±2.18a
Ald2	106-23-0	Citronellal	16.63	1131	89.4	32.2±6.29a	34.69±4.01a	36.01±2.93a
Ald4	2111-75-3	1-Cyclohexene-1-carboxaldehyde, 4-(1-methylethenyl)-	19.01	1271	81.4	35.88±0.00b	148.44±5.86a	46.18±5.99b
Ald6	112-31-2	Decanal	18.33	1200	89.1	ND	90.55±11.53b	159.2±19.09a
Ket1	76-22-2	Camphor	16.32	1114	88.7	20.38±3.26a	16.04±1.96a	19.72±1.88a
Ket2	464-48-2	Bicyclo[2.2.1]heptan-2-one, 1,7,7-trimethyl-, (1S)	16.32	1146	91.5	20.38±3.26a	16.04±1.96a	19.72±1.88a
Ket4	99-49-0	Carvone	19.5	1211	91	47.39±4.6b	84.09±1.11a	36.52±5.52b
Ket5	110-93-0	5-Hepten-2-one, 6-methyl-	10.62	961	89.4	3.13±0.00ab	4.49±0.95a	2.4±0.00b
Ket3	464-49-3	(+)-2-Bornanone	16.32	1121	89.9	ND	ND	18.63±2.17a
Phe1	89-83-8	Thymol	20.79	1293	86.4	66.8±2.62a	17.21±2.3.00c	42.42±6.39b
Phe2	499-75-2	Phenol, 2-methyl-5-(1-methylethyl)	20.97	1301	83.8	66.8±2.62a	36.95±2.76b	42.42±6.39b
Oth2	622-96-8	Benzene, 1-ethyl-4-methyl-	9.62	940	89.4	ND	ND	2.99±0.01a
Epo1	4959-35-7	Limonene oxide, trans	16.12	1121	88.8	18.68±3.63b	44.60±5.75a	13.71±1.22b
Epo2	13837-75-7	Limonene oxide, cis	16.11	1116	80.2	15.84±1.26a	ND	ND

Note: Data are expressed as mean ± standard deviation. ND indicates not detected. different superscript letters in the same row indicate significant differences ($P < 0.05$).
